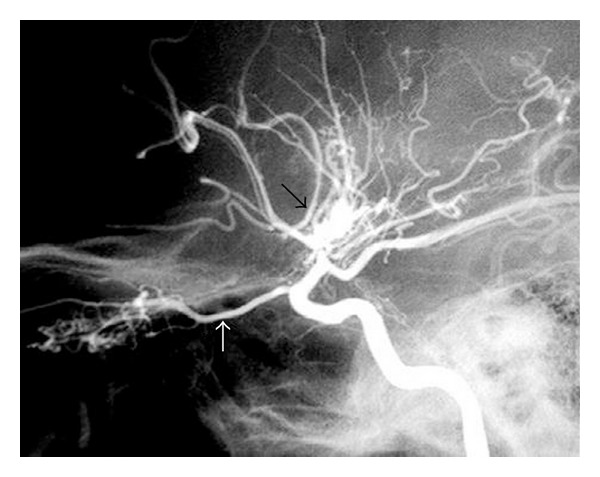# Moya Moya Disease in a Child: A Case Report

**DOI:** 10.1155/2011/329738

**Published:** 2011-09-18

**Authors:** Jagdish P. Goyal, Sanjeev S. Rao, Sangita Trivedi

**Affiliations:** Department of Pediatrics, Government Medical College & New Civil Hospital, Surat, Gujarat 395001, India

## Abstract

We report a case of 8-year-old female child who was admitted at our hospital with complaints of transient ischemic attacks and left-sided hemiparesis. On MR angiography, the child was diagnosed to have Moya Moya disease. Moya Moya disease is a rare cause of cerebral stroke in children. The patient was treated conservatively and referred to a higher centre for specific neurosurgery. Neurosurgical revascularization process leads to favourable outcome.

## 1. Introduction

Acute stroke is an infrequent disease of paediatric age group patients. Moya Moya is a rare cerebrovascular disease of unknown aetiology. We report a case of 8-year-old female child who presented with left-sided hemiplegia to a tertiary care hospital in India and diagnosed as Moya Moya disease. Cerebral revascularization surgery leads to favourable outcome.

## 2. Case History

An 8-year-old female child was admitted at our institute with complaints of weakness over left upper limb followed by lower limb for 20 days. There was also history of sudden fall 3 times probably indicating transient ischemic attacks. There was no history of fever, convulsion, head injury, and ear discharge. There was no history of delayed mile stones. There were no neurocutaneous markers or asymmetry of face. On neurological examination, gait was hemiplegic, tone was decreased over left side, power was 2/5 over left upper and lower limb, deep tendon reflexes were exaggerated, and planter was extensor over left side. Haematological and CSF examination was normal. MRI brain was suggestive of right sided hemiatrophy with frontal gliotic changes and subacute right occipital MCA territory/watershed zone infarct. MR angiography brain revealed generalized irregularity and beaded appearance in both posterior cerebral arteries (right > left). There were few Moya Moya collaterals detected in right basal ganglia region, which gives “puff of smoke” appearance (Figure 1). Reduced calibre of right CCA is also seen as compared to left side.

 We treated the patient conservatively with Glycerol 1 mL/kg/day and Aspirin 5 mg/kg/day. Patient showed slight improvement on power over left side (3/5). As our centre is not equipped with such kind of neurosurgery, so we referred the child to higher centre for surgery where cerebral revascularization surgery using encephaloduroarteriosynangiosis (EDAS) was done. The child showed marked improvement after surgery. Her hemiparesis was improved after 6 months, and repeat MRI was also found to be normal.

## 3. Discussion

Moya Moya disease is a rare disease characterized by multiple occlusions of the cerebral circulation with an unusual net like system of collaterals. In Japanese, Moya Moya means “hazy”. The disease derives its peculiar name from the angiographic appearance of cerebral vessels in the disease that resembles a “puff of smoke”. In children, the most common presentation is that of recurrent episodes of cerebral ischemia manifesting clinically as focal deficits, paresthesiae, and seizures [1]. Previously thought to be prevalent only in Japan, cases have now been reported from across the globe [2, 3]. However, majority of the cases are reported in Asia and other non-Caucasian regions [4].

The process of narrowing of cerebral vessels seems to be a reaction of brain blood vessels to a wide variety of external stimuli, injuries, or genetic defects. Conditions such as sickle cell anemia, neurofibromatosis-1, Down's syndrome, congenital heart defects, antiphospholipid syndrome, renal artery stenosis, and thyroiditis have been found to be associated with Moya Moya disease in the literature [5]. But more than half of the children seen with this disease have no cause for their Moya Moya syndrome. The process of blockage, once it begins, tends to continue despite any known medical management unless treated with surgery [6]. MRI not only reveals areas of infarctions, but also allows direct visualization of these collateral vessels as multiple small flow voids at the base of brain and basal ganglia. MR angiography is used to confirm the diagnosis and to see the anatomy of the vessels involved. It typically reveals the narrowing and occlusion of proximal cerebral vessels and extensive collateral flow through the perforating vessels demonstrating the classic puff of smoke appearance [7].

Acute management is mainly symptomatic and directed towards reducing elevated intracranial pressure, improving cerebral blood flow, and controlling seizures. Revascularization procedures are currently performed to increase the perfusion to the hypoxic brain tissue. The literature supports these procedures, and long-term favourable outcome has been reported in terms of improvement in symptoms and positive angiographic followups in all age groups [8]. Prognosis of patients with Moya Moya disease is found to be related to age and the type of presentation. Hemodynamic improvement after surgical procedures appears to be similar in all age groups. TIA and epileptiform clinical pictures have a better long-term outcome when compared to infarctions [9].

This case highlights the importance that early diagnosis and management leads to favourable outcome in children with Moya Moya disease.

## Figures and Tables

**Figure 1:**